# Effects of information involvement on subjective well-being during public health emergencies: the mediating roles of emotional regulation and social support

**DOI:** 10.3389/fpubh.2025.1490771

**Published:** 2025-03-19

**Authors:** Ke Zhang, Ting Jin, Yuanyuan Chen, Liwen Jiang, Yuchen Xie, Jing Wang

**Affiliations:** ^1^School of Communication, Soochow University, Suzhou, Jiangsu, China; ^2^City Culture and Communication College, Suzhou City University, Suzhou, Jiangsu, China

**Keywords:** public health emergencies, information involvement, emotional regulation, social support, subjective well-being

## Abstract

Subjective well-being is an important criterion to measure the quality of individual life. Based on social support theory and emotional regulation theory, this research tests the effects of individual and environmental factors on subjective well-being during public health emergencies. 1,488 valid samples were collected through an online questionnaire survey. The results show that: (1) Individuals’ perceived involvement of information related to public health emergency significantly influences their generalized anxiety and social media self-disclosure; (2) Generalized anxiety during public health emergency affects subjective well-being through emotional regulation and social expectation; (3) Social media self-disclosure during a public health emergency affects subjective well-being through social support and social expectation; (4) Social loneliness negatively moderates the effect of emotional regulation on subjective well-being, with lower loneliness strengthening this effect; (5) Social anxiety positively moderates the effect of social support on subjective well-being, with higher anxiety strengthening this effect. This study provides insights for the public to better cope with public health emergencies and improve their subjective well-being through adjusting their emotion and seeking social support.

## 1 Introduction

A public health emergency is an event that can cause harm to a person’s health or to the health of a community, including outbreaks, natural disasters, accidental releases of industrial chemicals, and intentional acts with biological, chemical, radiological, or nuclear agents (1). The WHO has also introduced the concept of a Public Health Emergency of International Concern (PHEIC), referring to major public health events that pose a global health risk, including COVID-19, Ebola, and the Zika virus. Due to the global impact of the COVID-19 pandemic, PHEIC have garnered significant academic attention (2). This study also focuses on outbreak-related public health emergencies.

The occurrence of public health emergencies is often accompanied by psychological crises in the general public. During the COVID-19 pandemic, the incidence of negative psychology in the global general population generally increased, namely, the incidence of anxiety was 50.9%, depression was 48.3%, and post-traumatic stress disorder was 53.8% (3). Public health emergencies not only directly affect the psychology of the public, but also the dissemination of related information will further aggravate the psychological response of the public. A great deal of rational and irrational information related to public health emergencies influences public sentiment. On the one hand, health information related to public health emergencies is the key for the public to understand the development of the events and make information decisions. On the other hand, psychological or emotional problems can arise when the amount of information the public receives exceeds their capacity to process it. The mental health status of the public will change with the development of emergencies (4). It is necessary to pay continuous attention to the mental health status of the public during and after public health emergencies.

Previous studies have primarily focused on the emergency management strategies and effect mechanisms on public health emergencies. After the outbreak of COVID-19, the relationship between the spread of pandemic-related information on social media and public health has gained attention. Social media quickly spreads information encompassing diverse topics and emotional expressions in public health emergencies (5). During the COVID-19 pandemic, online users eased their panic by seeking outbreak-related information, while also meeting their social needs through discussions with others (6). On the other hand, studies have revealed the existence of the “infodemic” phenomenon during public health emergencies, referring to the excessive spread of both accurate and false information, including conspiracy theories, fake news, and unproven cures, which can lead to confusion, heightened anxiety, and serious risks to public health and safety (7). For example, a study examined the antecedents and consequences of information overload during COVID-19, finding that overload impaired individuals’ information processing, leading to less effective prevention behaviors (8).

Subjective well-being is an important research direction and psychological quality in positive psychology. From a social point of view, subjective well-being is an important index and motivation to measure and promote social harmony. From the individual point of view, subjective well-being is also an important indicator of individual mental health evaluation. This large-scale outbreak of infectious diseases directly affects the public’s information processing and mental health, and further affects subjective well-being. Under public health emergencies, individual and environmental factors such as information involvement, social media self-disclosure, generalized anxiety during a public health emergency, emotional regulation style, social support, social expectation, social anxiety, social loneliness, and other factors jointly influence the subjective well-being of the public. Based on the background of public health emergencies, this study will explore the impact of public information involvement on psychology and behavior under such emergencies, and on this basis, study the influencing factors of subjective well-being. To further point out how to help the public better cope with emergencies, adjust emotions and improve subjective well-being, this paper puts forward specific suggestions.

Based on the research background above, we propose the following research questions: (1) In the context of public health emergencies, what impact does information involvement have on the psychology and behavior of the public? (2) In the context of public health emergencies, how does the psychology and behavior of the public, influenced by information involvement, further affect subjective well-being, and what is the underlying mechanism?

## 2 Theoretical framework and hypotheses

### 2.1 Public health emergency

Public health emergencies refer to major infectious disease outbreaks, mass diseases of unknown cause, major food and occupational poisoning, and other events that seriously affect public health that occur suddenly and cause or may cause serious damage to public health (9). Previous studies mostly focused on the emergency management, emergency mechanism and emergency treatment of public health emergencies themselves. However, with the development of information and communication technology, social media has gradually become an important channel for people to discuss events, share opinions and interact. Scholars are gradually realizing the role of social media in public health emergencies (10). Malecki et al. (11) proposed in their study that netizens can relieve panic by searching for information related to the novel coronavirus pandemic, and also meet their social needs by discussing the epidemic information with other netizens. However, some scholars have explained that the use of social media is not only beneficial but also harmful from another perspective. Pang et al. (12) built a theoretical model of the causes and consequences of social media overload in public health emergencies, and showed that information overload in such incidents will directly affect people’s cognitive and emotional responses, and then affect their social media coping behaviors.

### 2.2 Subjective well-being

Human well-being is defined as optimal mental functioning and refers to the subjective evaluation of well-being, which includes all judgments about the good and bad experiences in life (13). Subjective well-being relies on experiences that include both reflective cognitive judgments and emotional responses to life in the present moment. The study of subjective well-being has found scientific applications in psychology and other fields (14).

The current research also increasingly attaches importance to the impact on well-being, which contributes to physical and mental health, prevents mental illness, and even leads to longevity. People with higher levels of well-being are more creative and have better relationships with others (15). Since the outbreak of COVID-19, many studies have focused on the impact of public health emergencies on subjective well-being. Research shows that changes in the labor market caused by COVID-19 have harmed workers’ subjective well-being (16). Xie et al. (17) investigated the mental health status of students in Hubei Province, China, during home confinement due to the pandemic and found that they had increased and unexpected symptoms of depression and anxiety. With the rise of social networks, research on subjective well-being has also extended online. Zhang et al. (18) proposed the construction of e-social well-being, defined as an individual’s satisfaction with their social life in cyberspace.

### 2.3 Information involvement

Information involvement refers to the degree or extent to which an individual is related to or engaged with information in a specific context (19). It measures the level of interest, attention, and investment that an individual has toward specific information or topics. The level of information involvement depends on the importance, relevance, and attractiveness of the information object, as well as the individual’s cognitive, emotional, and behavioral engagement with that information (20). Higher information involvement indicates a stronger level of investment and attention from the individual on specific information or topics, while lower information involvement suggests relatively less attention or engagement from the individual toward that information (21).

The degree of public involvement in information related to public health emergencies not only includes the evaluation of the event but also includes the degree of attention and understanding of the event. Research on information involvement primarily focuses on information processing styles which play an important role in psychological adaptation (22). Information has the effect of emotional contagion. As for information involvement, some scholars believe that social interaction is a part of human daily life from the perspective of emotional infection, and emotional infection plays an important role in this interaction process (23, 24).

### 2.4 Generalized anxiety during public health emergency

Generalized anxiety refers to the fear of a danger that has not yet happened, which is a diffuse and negative emotional experience, which is closely related to the environment and individual stress level (25). After experiencing a disaster event, people will have a combination of psychological and physical symptoms, including initial panic, flight, helplessness and other emotions, and individuals’ perception of negative emotions will further aggravate negative coping behaviors (26). Such psychological discomfort will lead to more serious psychological disorders and pain in the long run. That is to say, after the release of the epidemic policy, the original anxiety will be relieved, but it will not disappear completely, and the individual anxiety will still exist.

In public health emergencies, the public is under a high level of pressure, and negative emotions such as anxiety and depression are significantly increased (27). More than 70% of the public feels a medium or above the level of pressure in the novel coronavirus pneumonia epidemic, which is significantly higher than the normal level (28). Studies have shown that there is a correlation between social media information exposure and mental health problems during the outbreak of COVID-19 in China, indicating that social media information exposure is associated with a high incidence of depression and anxiety, and users are highly exposed to the media environment due to concerns about themselves and the society, which may aggravate their anxiety (29). A survey during COVID-19 showed that media involvement was positively associated with death anxiety (23, 24). Based on the above literature review, we propose the first hypothesis:

*H*1: Individuals’ information involvement related to public health emergencies significantly affects generalized anxiety.

### 2.5 Self-disclosure

Social media can be an alternative medium for some people to practice self-disclosure, develop closer relationships and achieve their interpersonal goals (30). Self-Disclosure refers to individuals voluntarily sharing information about themselves with others (31), and from the perspective of social penetration theory (32), self-disclosure is described as the process of sharing different levels of information about themselves with others. Kasmani et al. (33) divide young people into three types of groups in terms of their level of self-disclosure on social media and its impact on their well-being: one is passive use of social media—self-disclosure avoidance; Active use of social media—positive emotional self-disclosure type; And active use of social media to express their happy and annoyed emotional states on social media. Emotion is information (34). When something causes individual emotional fluctuations, individuals need to express and vent their emotions through sharing behaviors, to find an emotional outlet to adjust themselves to a normal state.

Compared with traditional media, the interactive nature of network media is more likely to cause negative “emotional contagion” in disasters, which may make new media users experience more negative psychological effects, and watching more stressful content is also associated with more negative emotions and depression (35). Specifically, social media users may increase their pressure by watching and sharing uncensored media content, further aggravating public confusion and anxiety (36). The more attention the public pays to the relevant information reported by the media, the higher the perceived severity of the disease, and the more sensitive the public will be to the relevant information about the disease and have more concerns (37). This external environment can stimulate a person to actively participate in certain activities, such as sharing unverified information on social media (38).

Based on this, the second hypothesis is proposed:

*H*2: Individuals’ Information involvement related to public health emergencies significantly affects social media self-disclosure.

### 2.6 Emotional regulation

Emotional regulation is the process by which an individual exerts influence over what emotions they have when they occur, and how they are experienced and expressed (39). Emotional regulation can be both a means and a content, that is, the regulation of emotions themselves. In past research, emotional regulation has been widely used in the research of psychology and related disciplines, mainly involving the aspects of how people regulate their emotions, why they regulate their emotions, and what the consequences of emotional regulation are. Mauss et al. (40) pointed out that the more a person’s emotional behavior is in line with the individual’s internal emotional state, the better the social-communicative function of emotion will play. Positive emotional regulation can help people cope with the strong impact brought by stressful events. Emotional regulation is essentially a process of interaction between emotion and cognition, which not only includes the influence and drive of emotion on cognition but also involves cognition’s understanding and regulation of emotion. This study focuses on the latter perspective, focusing on the cognition and regulation of negative emotions in the context of public health emergencies.

The anxiety caused by public health emergencies will make the public take a series of coping measures, including physiological adjustment, which can be relieved by outdoor activities and physical exercise (41), and psychological adjustment, which requires adjustment of psychological state. Such as reassessing the situation and suppressing emotions (39).

Rumination, catastrophization, and self-blame were associated with poorer well-being, and positive reappraisal and refocus planning were positively correlated with both subjective and psychological well-being (42). Under the influence of COVID-19 stress events, higher levels of social support and positive emotional regulation strategies can effectively reduce the stress response level of college students (43). A study on the emotions of young women found that reappraisal in emotional regulation mediated the impact of attachment anxiety and avoidance on subjective well-being (44).

Based on this, the following hypothesis is proposed:

*H*3: Individuals’ generalized anxiety significantly affects subjective well-being through emotional regulation.

### 2.7 Social expectation

Social expectation refers to an individual’s reliance on social approval, as well as the avoidance or fear of social disapproval (45). It reflects the degree to which an individual depends on social recognition, encompassing both the need for social approval and the fear of social rejection (46). Social expectation can be conceptualized as a motivated and goal-directed behavior, wherein individuals exhibit a need for social approval, and the intensity of this need varies significantly across individuals (47). Individuals have a need to gain social approval, and the strength of this need varies among individuals.

Previous researches have demonstrated the relationship between anxiety, social expectation, and well-being. A study on children with anxiety disorders found that, compared to normal children, children with anxiety disorders have more negative social expectation (48). Rudolph et al. (49) pointed out that when people show high social expectation, the enhancement of self-social expectations will further buffer negative emotions. Research on tourists’ holiday expectations found that tourists waiting for a holiday experience higher well-being (50). However, other studies have found that among rural-to-urban migrant populations in China, individuals with higher social expectation tend to have lower well-being (51),

Based on this, the following hypothesis is proposed:

*H*4: Individuals’ generalized anxiety significantly affects subjective well-being through social expectation.

Stsiampkouskaya et al. (52) studied audience-oriented photo-sharing behavior on social media, and the results showed that, before selecting self-disclosure of photos on social media, participants in the study usually first considered the views of the audience and chose pictures that could arouse the interest of the audience for disclosure. When the feedback information received exceeds the expectation, Individuals will feel happy when receiving feedback. Therefore, individuals may be influenced by social expectation when they make self-disclosure, which is manifested in controlling others’ impression of them in a way of performance, to obtain others’ positive or pleasant evaluation of themselves (53). Such “performance” on social networks may be true or false. Research shows that self-disclosure in accordance with others’ expectations can achieve more social connections (54) and positive emotions.

Based on this, the hypothesis is proposed:

*H*5: Individuals’ social media self-disclosure significantly affects subjective well-being through social expectation.

### 2.8 Social support

Social support is a multi-dimensional structure with a range of definitions. Ke et al. (55) believe that social support can be divided into three categories: subjective support, objective support and support utilization. Liang et al. (56) also start from subjective support and believe that social support is an individual’s experience of being cared for, responded to and helped in a social group. It is believed that social support can bring warmth and understanding to individuals and meet their psychological needs. In this study, social support is summarized as the subjective support and objective support that a person has and can rely on, including the degree of utilization of these supports. This social support will provide reliable help when people face the stress of a public health emergency. Social support is often regarded as a social resource for individuals to cope with stress, and a large number of studies have also emphasized the impact of social relationship quality on mental health (57). Some studies have also emphasized that such external social resources can buffer the anxiety of college students through internal psychological resources (58). As for social support, there are three main theoretical models, namely the main effect model, buffer model and dynamic effect model. Combined with the research background, the social support referred to in this study is based on the main effect model, that is, the level of social support an individual has will directly affect subjective well-being.

In a socially supported relationship network, self-disclosure is necessary so that individuals can understand the status of their public relations with others. It is often the case that the more self-disclosure one makes with others, the more self-disclosure one will receive from others (59), which will strengthen the social relationship between two people. Graham et al. (60) argued that honest self-disclosure is important for effective social support and can only be aided in the context of open communication. Previous studies have also focused on the mediating role of social support between online self-disclosure and negative emotions. The results show that social support can be used as a good social resource to reduce loneliness and depression, etc., and the higher the level of social support, the more willing to self-disclose and improve positive emotions (61).

Based on this, the following hypothesis is proposed:

*H*6: Individuals’ social media self-disclosure significantly affects subjective well-being through social support.

### 2.9 Social loneliness

Social loneliness has become a common social psychological disease in modern society. It is a kind of negative emotion caused by not meeting one’s expectations of social connection. It is associated with many physical, mental or behavioral problems. Expressing loneliness can help people relieve negative emotions and gain social support (62). Three years after the outbreak of the novel coronavirus, ongoing infections around the world have increased loneliness and posed a potential threat to people’s physical and mental health. A large number of studies have shown that social loneliness can directly lead to anxiety and depression, as well as harm people’s physical health (63). Therefore, loneliness and its possible consequences make it particularly important to study social loneliness and related issues.

Loneliness will affect people’s ability to regulate emotions, and having social connections contributes to both upward and downward regulation of emotions. Individuals with higher levels of loneliness tend to reduce social sharing and increase rumination (64). The social baseline hypothesis holds that social connection encourages emotional regulation (65), such as reducing stress by sharing negative emotions with others, and regulating positive emotions by maximizing positive events. Individuals who create social environments on social networks, such as Twitter and Facebook, hope to alleviate loneliness and seek emotional support by sharing content or messages with others (66). Socially lonely people are considered to be withdrawn and unsociable, which means that sharing their emotions openly may reinforce the individual’s perception of loneliness and thus trap them in an emotional vicious cycle.

Therefore, people with a high level of social loneliness will not seek positive emotional regulation out of consideration of their image, and thus have emotional regulation disorders, which will further reduce their love for life and positive emotional experience, namely, their subjective well-being will be reduced.

Based on this, the following hypothesis is proposed:

*H*7: Social loneliness negatively moderates the effect of emotional regulation on subjective well-being, with lower social loneliness strengthening this effect.

### 2.10 Social anxiety

Social anxiety is the emotional experience of feeling uncomfortable, unnatural, nervous or even fearful when interacting with others, which can affect physical and mental health to varying degrees (67). Social anxiety ranges from reducing individual happiness, affecting academic studies and social interactions to depression and suicide (68).

Huang et al. (69) investigated the role of anxiety in the influence of social support on subjective well-being in their study, and the results showed that social support negatively predicted anxiety, while anxiety negatively predicted subjective well-being. As a negative emotion, social anxiety will hurt subjective well-being, and the pain individuals feel in interpersonal communication will, in turn, have a more negative impact on interpersonal relationships (70). However, people with sufficient social support can obtain more help from family members, friends, or classmates. Research has shown that social support can alleviate depression and anxiety (71), influence people’s psychological states, relieve anxiety, depression, and stress, and contribute to post-disaster psychological recovery (72). When affected by negative events, they will also have the resources and abilities to handle negative emotions. Therefore, in public health emergencies, individuals who already experience social anxiety are more prone to negative emotional experiences due to a lack of adequate social support. Based on this, the following hypothesis is proposed:

*H*8: Social anxiety negatively moderates the effect of social support on subjective well-being, with lower social anxiety strengthening this effect.

**Figure 1:**
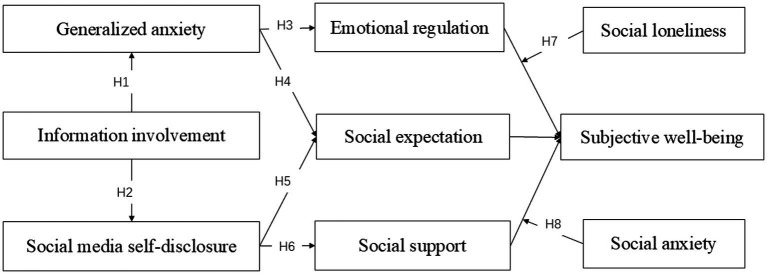
Conceptual model.

The conceptual framework is shown in Figure 1.

## 3 Methods

### 3.1 Measurement objectives

This study adopts a quantitative research method. We plan to achieve the following primary research objective through a questionnaire survey: examining the effects of information involvement on public psychology, especially subjective well-being, during public health emergencies;

And the following secondary research objectives: (1) Examining the impact of emotional regulation, social support, and social expectation as mediating variables on subjective well-being during public health emergencies; (2) Examining the effects of social anxiety and social loneliness as moderating variables on subjective well-being during public health emergencies.

Based on the research hypotheses, we propose the following research questions:

Q1: In public health emergencies, does higher information involvement lead to increased generalized anxiety compared to lower information involvement?

Q2: In public health emergencies, does higher information involvement lead to increased social media self-disclosure compared to lower information involvement?

Q3: Does generalized anxiety during public health emergencies influence subjective well-being through emotional regulation?

Q4: Does generalized anxiety during public health emergencies influence subjective well-being through social expectation?

Q5: Does social media self-disclosure during public health emergencies influence subjective well-being through social expectation?

Q6: Does social media self-disclosure during public health emergencies influence subjective well-being through social support?

Q7: Does social loneliness negatively moderate the effect of emotional regulation on subjective well-being, with lower social loneliness strengthening this effect?

Q8: Does social anxiety negatively moderate the effect of social support on subjective well-being, with lower social anxiety strengthening this effect?

### 3.2 Questionnaire design

In the context of the COVID-19 pandemic, we designed a questionnaire with 86 items to collect data. The questionnaire consists of two parts. The first part covers demographic statistics, including basic information such as gender, age, education level, monthly income, marital status, as well as extended information such as living conditions, health status, number of COVID-19 infections, and duration of social media use. The second part measures all the variables involved in the hypotheses.

All items were adopted from established scales. The questionnaire in this study used a 5-point Likert scale, where respondents rated each statement based on their views, with scores ranging from 1 (strongly disagree) to 5 (strongly agree). Before the formal data collection, we conducted a pilot test (*N* = 50). The overall Cronbach’s α coefficient of the pilot questionnaire was 0.893, indicating good reliability. However, the reliability of the information involvement and social expectation scales was relatively low in the individual scale assessments. As a result, the item wording for these two scales was revised in the formal survey, leading to acceptable reliability.

Reliability and validity tests were conducted on the formal survey. The Cronbach’s α coefficient was 0.813, with subscales above 0.6, indicating good reliability. The KMO value was 0.973 and *p* < 0.05, confirming acceptable validity.

The sources, number of items, and reliability of each scale are presented in Table 1.

**Table 1:** Measurement scales.

Variable	Resources	Number of items	Cronbach alpha
Information involvement	Zaichkowsky (75)	4	0.654
Generalized anxiety	Silva et al. (76)	7	0.882
Social media self-disclosure	Osatuyi et al. (77)	12	0.901
Social support	Zimet et al. (78)	12	0.673
Subjective well-being	Diener et al. (79)	17	0.813
Social loneliness	Wongpakaran et al. (80)	6	0.920
Social anxiety	Alkis et al. (81)	16	0.968
Social expectation	Stöber (82)	17	0.731
Emotional regulation	Gross and John (83)	10	0.852

### 3.3 Data collection

This study conducted questionnaire distribution through the Chinese online survey platform “Wenjuanxing,” and collected 1,619 questionnaires. The geographical location analysis of the questionnaire respondents showed that the survey data came from Jiangsu (24.71%), Liaoning (15.69%), Anhui (13.34%), Zhejiang (4.76%), Fujian (4.69%), Hubei (4.45%), Guangdong (4.08%), Shandong (3.27%), Sichuan (2.72%), Henan (2.66%), Hebei (2.21%), Shanghai (1.61%), and other regions. Then, some invalid questionnaires with too short answering time and obvious logical errors were eliminated, and 1,488 valid questionnaires were finally obtained, with an effective recovery rate of 91%.

According to the Seventh National Population Census Bulletin of China (2021), China’s total population is 1.443 billion (73). A significance level of *p* < 0.05 was set as the threshold for rejecting the null hypothesis. Assuming a precision of 3% (sampling error), a response rate of 50%, and a confidence level of 95%, it is estimated that at least 1,111 samples are required to evaluate the selected variables according to the minimum value calculation formula (74). In this survey, a total of 1,488 valid responses were received, which is more than the minimum sample size requirement and can reflect the characteristics of the overall sample to a certain extent.

## 4 Results

### 4.1 Descriptive analysis

A total of 1,488 valid questionnaires were collected, with 45.6% (*n* = 678) of respondents being male and 54.4% (*n* = 810) female, indicating a relatively balanced gender ratio. The majority fell within the 18–30 age group (37.1%, *n* = 552), followed by 31–43 years (30.0%, *n* = 446) and 44–56 years (28.0%, *n* = 416).

Regarding monthly income, 32.2% (*n* = 479) earned 5,000–10,000 yuan, while 30.9% (*n* = 460) earned 2,000–5,000 yuan. In terms of marital status, 38.8% (*n* = 578) were unmarried, while 55.3% (*n* = 823) were married. 31.8% (*n* = 473) lived alone, whereas 68.2% (*n* = 1,015) lived with others.

For COVID-19 infection history, 54.2% (*n* = 806) were infected once, 17.1% (*n* = 255) twice, and 2.2% (*n* = 32) three or more times. In terms of daily social media usage, 34.3% (*n* = 511) spent 1–3 h per day, 31.7% (*n* = 471) spent 3–5 h, and 22.2% (*n* = 330) spent more than 5 h. The demographic characteristics are summarized in Table 2.

**Table 2:** Demographic characteristics (*N* = 1,488).

Items	Options	Frequency	Percentage (%)
Gender	Male	678	45.6
	Female	810	54.4
Age	Under 18 years old	36	2.4
	Ages 18 to 30	552	37.1
	Age 31 to 43	446	30
	Ages 44 to 56	416	28
	Ages 57 to 69	27	1.8
	Age 70 and older	11	0.7
Educational background	High school or less	187	12.6
	Junior College	325	21.8
	Undergraduate college	740	49.7
	Master’s students and above	236	15.9
Monthly income	Less than 1,000 yuan	99	6.7
	1,000 to 2000 yuan	132	8.9
	2,000 to 5,000 yuan	460	30.9
	5,000 ~ 10,000 yuan	479	32.2
	10,000 to 20,000 yuan	207	13.9
	More than 20,000 yuan	111	7.5
Marital status	Unmarried	578	38.8
	Married	823	55.3
	Divorced or widowed	87	5.8
Residence situation	Living alone	473	31.8
	Non-solitary	1,015	68.2
Number of COVID-19 infections	0 infections	395	26.5
	1 time	806	54.2
	2 times	255	17.1
	3 times and more	32	2.2
Duration of social media use	Under 1 h	176	11.8
	1 to 3 h	511	34.3
	3 to 5 h	471	31.7
	5 + hours	330	22.2

As shown in Table 2, the public believed that epidemic information had a relatively significant impact on their lives, and their generalized anxiety level was high. They held positive views on their self-disclosure on social media, social support, subjective well-being, social expectation, and emotional regulation. Their perception of social loneliness and social anxiety was relatively low.

### 4.2 Hypothesis paths test

Before analyzing the influence mechanism among variables, Pearson correlation analysis was conducted for each variable involved in this study. The results showed that all variables in the conceptual framework were significantly correlated with each other, as shown in Table 3.

**Table 3:** Correlation coefficients.

Variables	1	2	3	4	5	6	7	8
Information involvement (1)								
Generalized anxiety (2)	518**							
Social media self-disclosure (3)	469**	506**						
Social support (4)	306**	221**	156**					
Subjective well-being (5)	441**	353**	343**	441**				
Social loneliness (6)	−524**	−381**	−354**	−443**	−703**			
Social expectation (7)	444**	332**	272**	343**	428**	−348**		
Social anxiety (8)	−511**	−458**	−471**	−326**	−644**	798**	−281**	
Emotional regulation (9)	528**	458**	396**	385**	491**	−378**	846**	−364**

*p < 0.05, **p < 0.01.

This study conducted three linear regression analyses to examine the effects of information involvement on generalized anxiety, social media self-disclosure, and subjective well-being, with demographic variables included as control variables. The linear regression results are shown in Table 4.

**Table 4:** Results of regression analysis.

Variable	Generalized anxiety	Social media self-disclosure	Subjective well-being
	M1	M2	M3
Control variable			
Age 1	0.195***	0.108*	−0.014
Age 2	0.205***	0.091	−0.197*
Monthly income 1	0.129*	0.104	0.295*
Monthly income 2	0.107	0.067	0.277*
Monthly income 3	0.429	0.025	0.078
Marriage 1	0.015		
Number of infections 1	−0.189***	−0.225***	0.194*
Number of infections 2	0.058	0.004	0.267*
Education 1		0.146*	
Education 2		0.186**	
Education 3		0.144*	
Residence situation			0.196*
Duration of use 1			−0.390**
Duration of use 2			−0.535***
Duration of use 3			−0.579***
Main effect			
Information involvement	0.593***	0.480***	0.921***
F	72.786	44.896	34.598
R^2^	0.307	0.251	0.220
△R^2^	0.038	0.030	0.025

Age 1 = “31 to 43,” age 2 = “44 +,” monthly income 1 = “2,000 to 5,000 yuan,” monthly income 2 = “5,000 to 10,000 yuan,” Monthly income 3 = “more than 10,000 yuan,” marriage 1 = “married,” number of infections 1 = “once,” number of infections 2 = “twice or more,” education 1 = “junior college,” education 2 = “college bachelor,” Education 3 = “Master’s degree or above,” duration of use 1 = “1–3 h,” duration of use 2 = “3–5 h,” duration of use 3 = “more than 5 h.” ***p < 0.001,**p < 0.01, *p < 0.05.

The results of the linear regression are shown in Table 4:

Generalized anxiety (M1): The model was significant (*F* = 72.786, *p* < 0.001), with an influence coefficient of B = 0.593 (*t* = 24.126, *p* < 0.001), indicating that higher information involvement leads to higher generalized anxiety, supporting H1.

Social media self-disclosure (M2): The model was significant (*F* = 44.896, *p* < 0.001), with an influence coefficient of B = 0.480 (*t* = 20.542, *p* < 0.001), showing that higher information involvement increases social media self-disclosure, supporting H2.

Subjective well-being (M3): The model was significant (*F* = 34.598, *p* < 0.001), indicating that higher information involvement is associated with higher levels of subjective well-being.

### 4.3 Mediating effects test

We used the Bootstrap method to test the mediating effects of emotional regulation, social expectation, and social support. The PROCESS V3.5 plug-in in SPSS was employed with a sample size of 5,000 and a 95% confidence interval. The results are shown in Table 5.

**Table 5:** Results of mediating effects.

Paths	Effect	SE	LLCI	ULCI	Proportion of mediating effect
Generalized anxiety→Emotional regulation →Subjective well-being	0.1909	0.0251	0.1588	0.2260	54.03%
Generalized anxiety→Social expectation →Subjective well-being	0.1161	0.0128	0.0920	0.1426	32.86%
Social media self-disclosure→Social expectation →Subjective well-being	0.0984	0.0126	0.0743	0.1236	28.66%
Social media self-disclosure→Social support →Subjective well-being	0.0619	0.0120	0.0378	0.0851	18.03%

The test on the mediating role of emotional regulation does not include 0 in the confidence interval (LLCI = 0.1588, ULCI = 0.2260), indicating that there is a mediating effect of emotional regulation on generalized anxiety and subjective well-being, accounting for 54.03%. Specifically, the higher level of generalized anxiety, the stronger degree of emotional regulation, and thus the subjective well-being was further improved, assuming that H3 was supported.

The test on the mediating role of social expectation does not include 0 in the confidence interval (LLCI = 0.0920, ULCI = 0.1426), indicating that social expectation has a mediating effect between generalized anxiety and subjective well-being, accounting for 32.86%. The higher level of generalized anxiety, the higher level of social expectation, and thus the subjective well-being was further improved, assuming hypothesis H4 was supported.

The test on the mediating role of social expectation does not include 0 in the confidence interval (LLCI = 0.0743, ULCI = 0.1236), indicating that social expectation has a mediating effect between social media self-disclosure and subjective well-being, accounting for 28.66%. The higher level of social media self-disclosure, the higher level of social expectation, and thus the subjective well-being was further improved, assuming hypothesis H5 was supported.

The test on the mediating role of social support does not include 0 in the confidence interval (LLCI = 0.0378, ULCI = 0.0851), indicating that social support has a mediating effect between social media self-disclosure and subjective well-being, accounting for 18.03%. The higher level of social media self-disclosure, the higher level of social support, and thus the subjective well-being was further improved, assuming hypothesis H6 was supported.

### 4.4 Moderation effects test

Hierarchical regression analysis was used to test the moderating effects of social loneliness and social anxiety. The Process V3.5 plugin in SPSS 26.0 was then applied, selecting Model 1 and the Bootstrap method with a sample size of 5,000 and a 95% confidence interval. The moderating effect results are shown in Table 6.

**Table 6:** Results of moderating effects.

	Variables	Unstandardized coefficient	Standard error	Coefficient of standardization	*t*	*p*	R^2^	△R^2^
Model 1	Emotional regulation	0.660	0.047	0.263	14.030	0.000		
	Social loneliness	0.971	0.030	0.604	32.219	0.000		
Model 2	Emotional regulation	1.116	0.129	0.444	8.631	0.000	0.557	0.004 (*p* = 0.004)
	Social loneliness	0.381	0.159	0.237	2.399	0.017		
	Emotional regulation X Social loneliness	0.155	0.041	0.348	3.780	0.000		
Model 1	Social support	0.687	0.053	0.259	13.001	0.000		
	Social anxiety	0.880	0.031	0.560	28.135	0.000		
Model 2	Social support	0.340	0.170	0.128	2.001	0.046	0.475	0.002 (*p* = 0.032)
	Social anxiety	1.321	0.208	0.841	6.343	0.000		
	Social support X Social anxiety	0.117	0.055	0.272	2.145	0.032		

The moderating effect of social loneliness on the relationship between emotional regulation and subjective well-being was tested. After adding the interaction term (emotional regulation × social loneliness), the model fit improved, with the adjusted R^2^ reaching 0.004 (*p* < 0.05), indicating a significant moderating effect, assuming H7 was supported.

**Figure 2:**
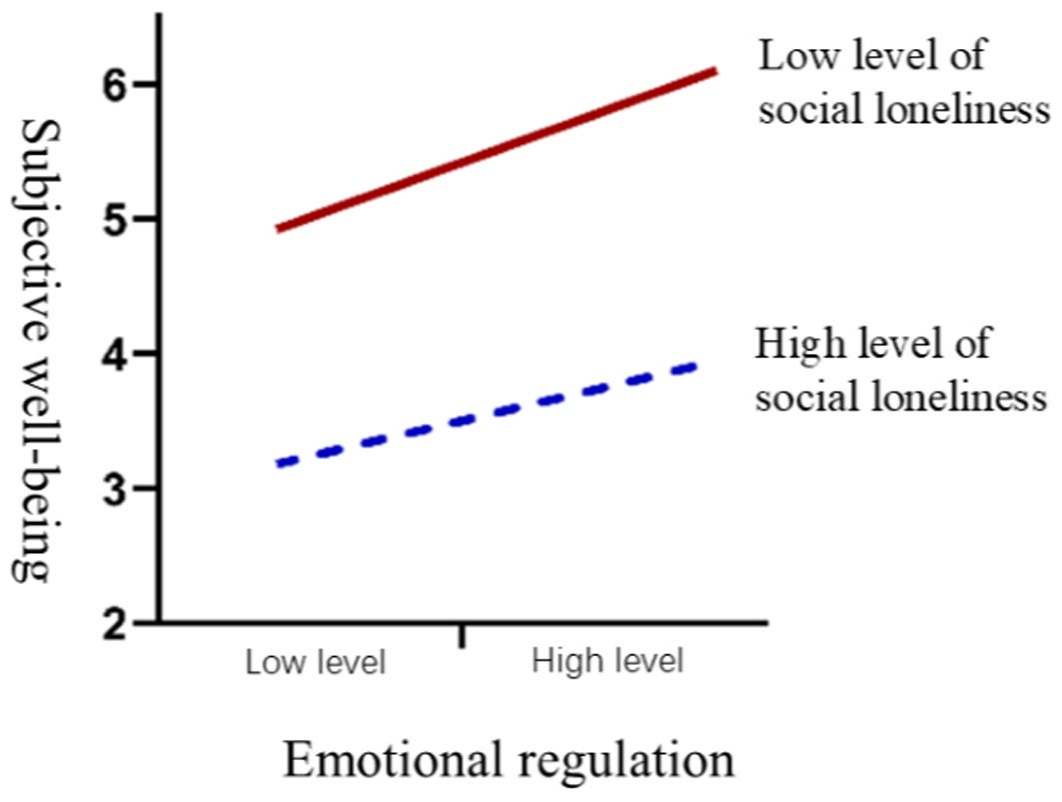
Moderating effect of social loneliness between emotional regulation and subjective well-being.

This suggests that social loneliness weakens the positive impact of emotional regulation on subjective well-being. For individuals with high social loneliness, the positive impact of emotional regulation on subjective well-being is weaker compared to those with low social loneliness. The simple slope plot for the moderating effect of social loneliness is shown in Figure 2.

The moderating effect of social anxiety on the relationship between social support and subjective well-being was tested. After adding the interaction term (social support × social anxiety), the model fit improved, with the adjusted R^2^ reaching 0.002 (*p* < 0.05), indicating a significant moderating effect, assuming H8 was supported.

**Figure 3:**
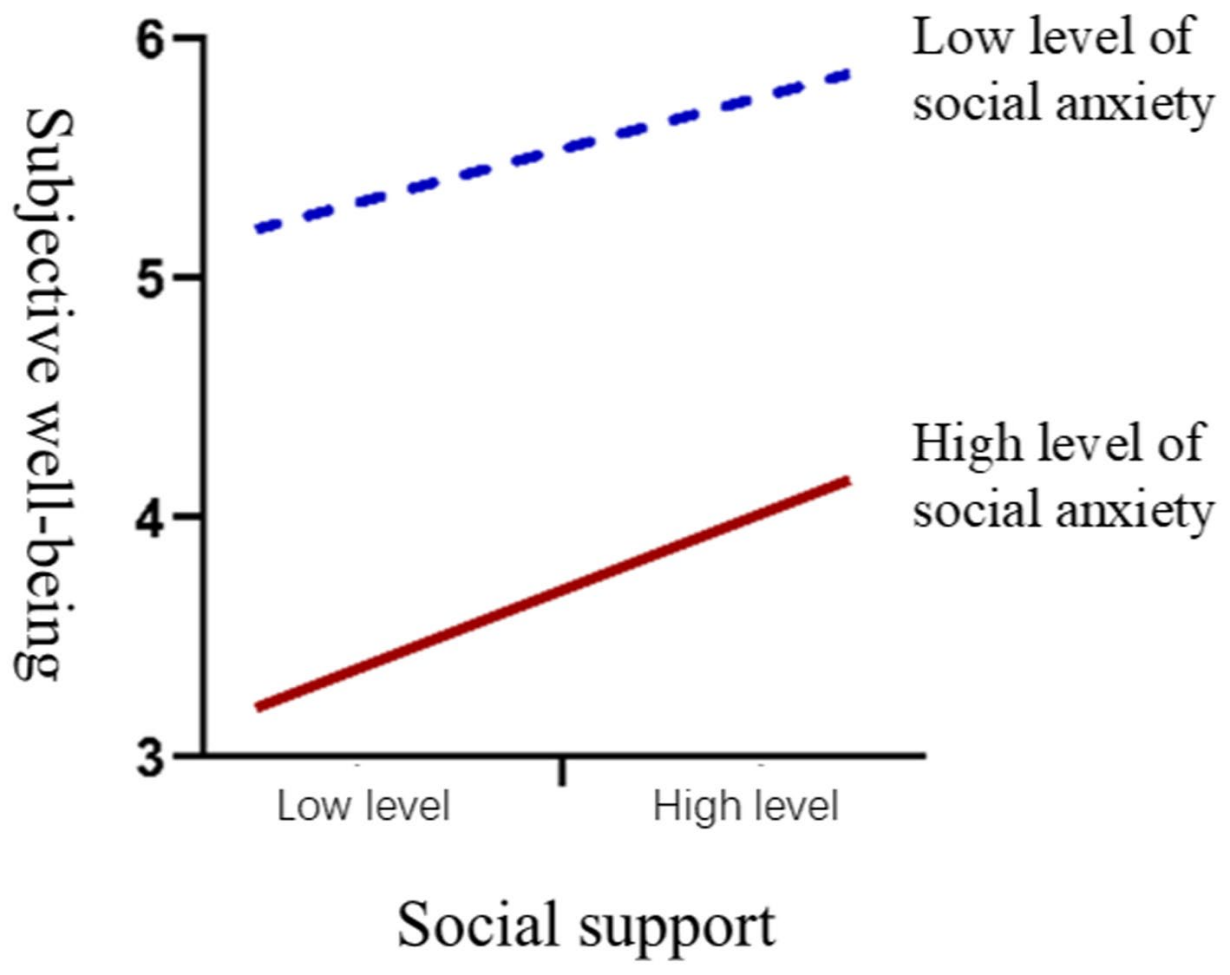
Moderating effect of social anxiety between social support and subjective well-being.

This suggests that social anxiety weakens the positive impact of social support on subjective well-being. For individuals with high social anxiety, the positive impact of social support on subjective well-being is weaker compared to those with low social anxiety. The simple slope plot for the moderating effect of social anxiety is shown in Figure 3.

All hypothesis test results are presented in Table 7.

**Table 7:** Hypothesis test results.

Hypotheses	Results
H1: Individuals’ information involvement related to public health emergencies significantly affects generalized anxiety.	Supported
H2: Individuals’ Information involvement related to public health emergencies significantly affects social media self-disclosure.	Supported
H3: Individuals’ generalized anxiety significantly affects subjective well-being through emotional regulation.	Partially supported
H4: Individuals’ generalized anxiety significantly affects subjective well-being through social expectation.	Partially supported
H5: Individuals’ social media self-disclosure significantly affects subjective well-being through social expectation.	Supported
H6: Individuals’ social media self-disclosure significantly affects subjective well-being through social support.	Supported
H7: Social loneliness negatively moderates the effect of emotional regulation on subjective well-being, with lower social loneliness strengthening this effect.	Supported
H8: Social anxiety negatively moderates the effect of social support on subjective well-being, with lower social anxiety strengthening this effect.	Supported

## 5 Discussion

### 5.1 Information involvement affects public psychology and behavior

The findings of this study support H1, indicating that individuals’ information involvement related to public health emergencies significantly affects generalized anxiety. This result is consistent with previous research showing that increased exposure to media during public health crises, like the COVID-19 pandemic, is associated with higher levels of anxiety and depression (27, 29). As individuals consume more information about the emergency, they may become overwhelmed, leading to heightened feelings of fear and anxiety, which confirms the proposed relationship between information involvement and generalized anxiety.

These results align with prior studies that highlight how media exposure during crises can intensify anxiety, particularly through social media platforms that amplify emotional responses (23, 24). The findings suggest that individuals’ heightened engagement with information during public health emergencies may exacerbate their anxiety levels. This highlights the importance of managing information dissemination to avoid overloading the public, which could further increase stress and anxiety.

The results of this study support H2, showing that individuals’ information involvement related to public health emergencies significantly affects social media self-disclosure. This finding aligns with existing literature suggesting that the more involved individuals are with information during a crisis, the more likely they are to share their thoughts and emotions on social media (33). As individuals are exposed to heightened levels of information during public health emergencies, they often seek an emotional outlet through self-disclosure, either to process their anxiety or to connect with others who share similar experiences (30). This result emphasizes the role of social media as a platform not only for information exchange but also as a mechanism for emotional expression and coping during stressful situations.

Additionally, the findings corroborate research indicating that media consumption, particularly during high-stress events, can lead to emotional contagion, where negative emotions are shared and amplified within online communities (35). The more information individuals consume related to the public health crisis, the more likely they are to express their emotions, whether positive or negative, on social media (36). This study highlights how the nature of modern media can stimulate greater emotional self-disclosure, which could be a coping strategy for individuals trying to manage the anxiety and confusion stemming from public health emergencies. Social media, thus, serves as both a platform for emotional release and a source of support during times of crisis.

### 5.2 Mediating role of social expectation, social support and emotional regulation

The findings of this study support H3, demonstrating that generalized anxiety significantly affects subjective well-being through emotional regulation. This result is consistent with previous research, which emphasizes the role of emotional regulation in mitigating the negative impacts of anxiety on well-being (42). Specifically, positive emotional regulation strategies, such as reappraisal, are shown to help individuals cope with anxiety induced by public health emergencies. Additionally, these findings align with studies that suggest emotional regulation can buffer stress and improve well-being during crises like the COVID-19 pandemic (43). Therefore, promoting effective emotional regulation strategies could be a key factor in enhancing individuals’ psychological resilience and improving their subjective well-being in the context of public health emergencies.

The findings of this study support H4, showing that generalized anxiety significantly influences subjective well-being through social expectation. The findings reveal that as generalized anxiety increases, individuals’ social expectation also rise, which further enhances their subjective well-being. Previous research has suggested that individuals with anxiety often rely on social approval to regulate their emotional state (48), a phenomenon confirmed by this study. Specifically, higher social expectation helps individuals alleviate anxiety and boost their well-being (50). The moderating role of social expectation is also supported by the literature. For instance, Rudolph et al. (49) found that when individuals receive higher social recognition, the increase in social expectation further alleviates negative emotions and improves psychological health. However, some studies also suggest that excessively high social expectation can create pressure, affecting individual well-being (26, 51). Therefore, the findings of this study indicate that the process by which generalized anxiety affects subjective well-being through social expectation may be influenced by individual cultural backgrounds and social environments, highlighting the complexity of moderating mechanisms in different social contexts.

The findings of this study support H5, showing that social media self-disclosure impacts subjective well-being through social expectation. Previous studies have found that people often choose what to share on social media based on what will interest their audience, which can lead to more social connections and improved well-being (52). Additionally, when individuals disclose in a way that matches social expectation, they are more likely to receive positive feedback, boosting their emotional health (53). Our study also confirms that aligning self-disclosure with social expectation leads to more positive emotional responses and greater well-being. This highlights how social media platforms, as places for self-expression, help improve emotional well-being by offering opportunities for validation and connection, especially during challenging times.

### 5.3 Moderating role of social loneliness and social anxiety

The findings of this study support H7, which suggests that social loneliness negatively moderates the effect of emotional regulation on subjective well-being. Individuals with high levels of social loneliness often struggle to regulate their emotions effectively, as they tend to withdraw from social interactions and avoid emotional sharing (64). This isolation prevents them from receiving emotional support, which is essential for positive emotional regulation (65). As a result, these individuals are more likely to experience a decline in their subjective well-being. In contrast, individuals with lower levels of social loneliness are more open to social interactions, which helps them regulate their emotions and improve their well-being (66). This finding emphasizes the importance of addressing loneliness and fostering social connections, especially during stressful events, to enhance emotional regulation and overall well-being.

The results of this study support Hypothesis H8, showing that social anxiety negatively moderates the effect of social support on subjective well-being. Specifically, individuals with lower levels of social anxiety experience a stronger positive impact from social support, while those with higher levels of social anxiety benefit less. This finding is consistent with previous research, which suggests that social anxiety reduces the effectiveness of social support in improving well-being (69, 70). When individuals experience high social anxiety, they may struggle to engage with their support networks, limiting the positive impact of social support on their mental health and well-being (68). Therefore, addressing social anxiety could be a key factor in enhancing the benefits of social support.

### 5.4 Theoretical contribution

This study explored the influence of information involvement on public psychology and behavior during public health emergencies, offering new insights into the psychological processes that shape individual responses in such contexts. By examining how information involvement affects generalized anxiety, social media self-disclosure, and subjective well-being, this research contributes to the theoretical understanding of how information processing during crises impacts emotional regulation, social expectation, and social support.

One significant theoretical contribution of this research is the finding that generalized anxiety, contrary to some traditional models, does not always lead to negative outcomes in terms of subjective well-being. Rather, it can act as a motivator for emotional regulation and self-disclosure, which, in turn, can enhance well-being under certain conditions. This challenges existing assumptions that anxiety is purely detrimental to well-being and introduces a more complex understanding of how anxiety, when managed effectively, can lead to positive psychological outcomes.

In conclusion, this study explores the dynamic relationship between information processing strategies and psychological factors in the context of public health emergencies, and develops a model of subjective well-being from the perspective of how individuals respond to such crises. The findings provide valuable theoretical insights and practical support for further exploration on how the dynamics of subjective well-being evolve across different cultural, social, and crisis contexts.

### 5.5 Practical implications

The research findings provide insights for different public health stakeholders on how to handle information during public health emergencies.

Public health authorities should focus on ensuring clear, accurate, and timely communication to reduce public anxiety. Providing reliable and emotionally supportive information through trusted platforms, such as government websites or health organization apps, can help mitigate negative psychological effects.

Social media platforms should focus on enhancing users’ information involvement to increase their willingness to self-disclose and reduce anxiety. By providing interactive social support and positive content, platforms can strengthen users’ sense of psychological safety and belonging. Additionally, avoiding the excessive spread of negative information and actively guiding users to maintain a healthy mindset can improve their subjective well-being.

Individuals should actively regulate their emotions and adjust their mindset to manage anxiety during a public health emergency. They can also engage in self-disclosure on social media to gain social support, while ensuring that the information they consume and share promotes well-being rather than increasing anxiety.

### 5.6 Limitations and suggestions for future research

While this study has verified the existing hypotheses, identified the status quo of subjective well-being after public health emergencies, and constructed an empirical impact model, some limitations remain.

First, this study focuses on public health emergencies caused by outbreaks, specifically in China, which makes the research context relatively narrow. Future studies can broaden this context by conducting cross-cultural comparisons to explore differences in psychological factors across cultures. For example, research could examine how social expectation influence subjective well-being in different cultural backgrounds. Additionally, future studies could focus on other types of public health emergencies to further investigate the relationship between information processing strategies and psychological factors, expanding the applicability of the research.

Second, this study primarily used survey data to explore the effects of different variables, which resulted in a relatively single method of analysis and lacks specific explanations for some of the findings. Future research could incorporate qualitative methods, such as interviews, to provide a more in-depth exploration of the relationship between information processing strategies and psychological factors. For instance, it could explain the specific psychological processes that lead to generalized anxiety improving subjective well-being through mediating variables.

Lastly, this study is cross-sectional in nature, which limits the ability to observe changes over time. Future research could implement longitudinal studies to track how the relationships between information involvement, anxiety, and subjective well-being evolve over time. Longitudinal research would provide insights into how these dynamics unfold in response to prolonged public health crises and could offer a deeper understanding of the long-term psychological impacts of such events.

## Data Availability

The original contributions presented in the study are included in the article/supplementary material, further inquiries can be directed to the corresponding author.
